# The Impact of Incentives on Data Collection for Online Surveys: Social Media Recruitment Study

**DOI:** 10.2196/50240

**Published:** 2024-07-04

**Authors:** Jessica Sobolewski, Allie Rothschild, Andrew Freeman

**Affiliations:** 1 RTI International Research Triangle Park, NC United States

**Keywords:** social media, online survey recruitment, incentive, experiment, online surveys, Facebook, Instagram, data collection, users, cost, social media recruitment, survey

## Abstract

**Background:**

The use of targeted advertisements on social media platforms (eg, Facebook and Instagram) has become increasingly popular for recruiting participants for online survey research. Many of these surveys offer monetary incentives for survey completion in the form of gift cards; however, little is known about whether the incentive amount impacts the cost, speed, and quality of data collection.

**Objective:**

This experiment addresses this gap in the literature by examining how different incentives in paid advertising campaigns on Instagram for completing a 10-minute online survey influence the response rate, recruitment advertising cost, data quality, and length of data collection.

**Methods:**

This experiment tested three incentive conditions using three Instagram campaigns that were each allocated a US $1400 budget to spend over a maximum of 4 days; ads targeted users aged 15-24 years in three nonadjacent designated market areas of similar size to avoid overlapping audiences. Four ad creatives were designed for each campaign; all ads featured the same images and text, but the incentive amount varied: no incentive, US $5 gift card, and US $15 gift card. All ads had a clickable link that directed users to an eligibility screener and a 10-minute online survey, if eligible. Each campaign ran for either the full allotted time (4 days) or until there were 150 total survey completes, prior to data quality checks for fraud.

**Results:**

The US $15 incentive condition resulted in the quickest and cheapest data collection, requiring 17 hours and ad spending of US $338.64 to achieve 142 survey completes. The US $5 condition took more than twice as long (39 hours) and cost US $864.33 in ad spending to achieve 148 survey completes. The no-incentive condition ran for 60 hours, spending nearly the full budget (US $1398.23), and achieved only 24 survey completes. The US $15 and US $5 incentive conditions had similar levels of fraudulent respondents, whereas the no-incentive condition had no fraudulent respondents. The completion rate for the US $15 and US $5 incentive conditions were 93.4% (155/166) and 89.8% (149/166), respectively, while the completion rate for the no-incentive condition was 43.6% (24/55).

**Conclusions:**

Overall, we found that a higher incentive resulted in quicker data collection, less money spent on ads, and higher response rates, despite some fraudulent cases that had to be dropped from the sample. However, when considering the total incentive amounts in addition to the ad spending, a US $5 incentive appeared to be the most cost-effective data collection option. Other costs associated with running a campaign for a longer period should also be considered. A longer experiment is warranted to determine whether fraud varies over time across conditions.

## Introduction

The use of targeted paid advertisements on social media platforms (eg, Facebook and Instagram) has become a popular method of recruiting participants for public health and medical research studies [1-3]. The broad user base of these platforms makes social media recruitment an advantageous tool for reaching large and diverse populations online [4]. Additionally, many studies have found that using targeting tools on these platforms to reach narrower audiences can be a cost-effective and time-efficient way to recruit populations who are typically undersampled, especially when compared to more traditional recruitment methods such as in-person intercept or online panels [5,6]. Often, studies using social media recruitment methods provide respondents with a monetary incentive, typically in the form of a gift card, as to compensate for completing the survey [2,6]. Previous research has shown that the presence and amount of an incentive impact various factors of data collection, including the total recruitment cost, length of data collection, and overall quality of data [7,8].

Several studies have examined the potential impact of varying incentive amounts, forms (eg, cash, gift card, reward points), and distribution methods (eg, immediate vs delayed, conditional vs nonconditional) on a variety of outcomes, including response rate and sample representativeness [7]. However, no research to date has examined these relationships in a study with participants recruited through social media platforms; rather, studies in this field frequently referenced online panels and email distribution lists as the primary modes of digital recruitment. A recent metareview identified 25 studies that examined the impact of incentives on response rates for web-based surveys [7,9]. These studies collectively demonstrated that, compared to no incentives, financial incentives (including monetary prizes, gift cards, and prize lotteries) were all associated with increased response rates to web-based surveys. Additional studies corroborate these findings, showing that higher incentives are associated with greater participation and response rates [9-14]. Another recent study found that a guaranteed incentive resulted in better retention rates among youth and young adult respondents compared to a lottery incentive [15].

Fewer studies have examined the impact of incentives on alternate outcomes such as data quality, data collection length, and recruitment costs. A 2019 study by Berzofky et al [16] analyzed the interactions between incentive amount, recruitment time, nonresponse, sample representativeness related to the population study of interest, and precision of survey estimates, and found that smaller incentives were often associated with lengthy data collection times, less representativeness, and less precise estimates overall. One study found that incentives were associated with more fraudulent respondents compared to a nonincentive control group, whereas larger incentives did not increase the rates of fraudulent responses [17]. Additional studies have also explored the impact of varying incentive amounts and forms on specific populations of interest such as applicants to a not-for-profit service organization (determined to be altruistic prosocial individuals) [18], college students [16], and medical providers [19].

This study addresses a gap in the literature by performing an experiment that examined how different incentive amounts influence response rate, data quality, length of data collection, and recruitment costs, all in the context of recruiting study participants through paid advertisements on social media. Researchers are increasingly using this methodology, but limited knowledge is available on how specific incentive amounts influence data collection processes and outcomes. To address this gap, this study specifically examined how advertising a US $5 incentive, US $15 incentive, and no incentive to youth and young adults (15 to 24 years of age) on Instagram for completion of an online survey impacted various data collection outcomes.

## Methods

### Study Design

To determine the impact of incentive amount on the response rate, recruitment costs, data quality, and recruitment time, we ran three separate paid social media campaigns on Instagram, one for each incentive condition. Each campaign was allocated a total budget of US $1400 to spend on ads over a maximum of 4 days; campaigns ran for either the full allotted time or until reaching 150 total survey completes, prior to data quality checks for each condition.

### Study Population

For each condition, we used targeting features on the platform to disseminate ads to users aged 15-24 years in three different designated market areas (DMAs) of similar combined size. All included DMAs were nonadjacent to avoid overlap in participants across conditions. We determined the relative size of the target population in each DMA using the “estimated audience size” metric in Meta Ads Manager (the platform used to manage Instagram advertisements and campaigns); this feature estimates the number of social media accounts that meet the targeting and ad placement criteria selected by advertisers. The nonadjacent DMAs were categorized into three groups based on their potential audience sizes; one DMA from each group was then randomly assigned to each condition to ensure each campaign had the potential to reach a similar number of accounts. Table 1 outlines the DMAs used for each condition along with their audience size, based on Meta estimates.

**Table 1:** Selected designated market areas (DMAs) and their estimated audience size among Instagram users aged 15-24 years assigned to each incentive condition.

Characteristic	No incentive	US $5 incentive	US $15 incentive
DMAs selected for targeting	Atlanta, GA; Phoenix (Prescott), AZ; Minneapolis-St. Paul, MN	Washington, DC (Hagerstown); Denver, CO; Seattle-Tacoma, WA	Boston, MA; Tampa-St. Petersburg, FL; San Francisco, CA
Estimated target audience size	2,500,000-2,900,000	2,300,000-2,700,000	2,500,000-3,000,000

### Survey Recruitment

**Figure 1:**
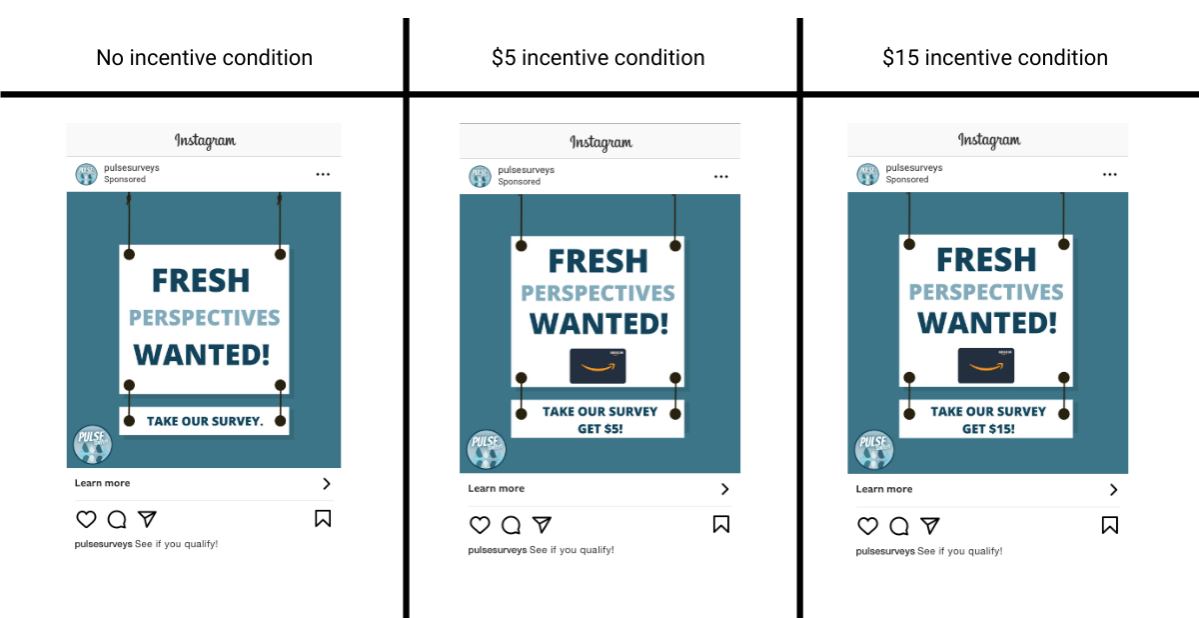
Examples of social media advertisements used on Instagram for each incentive condition.

We used a pixel to optimize all three campaigns for conversions by placing a custom conversion event on the back end of the screener to keep a record of users who screened as eligible. Instagram used these collected metadata about eligible users from the pixel to distribute ads to similar users who may likely be eligible. We developed four paid ad creatives for each campaign; all featured the same images and text, but the incentive amount was varied (see examples in Figure 1). All ads directed potentially eligible social media users to click a link that navigated to an online screener hosted in Qualtrics. Users who were determined to be eligible (15-24 years old and living in one of the three DMAs in their incentive condition) were offered to complete the full survey, which was estimated to take approximately 10 to 15 minutes, for the advertised incentive amount. The full survey included questions about preferences and behavior related to social media platforms, online surveys, and social media ads; these survey results are not presented in this paper.

The eligibility screener included several programmed fraud prevention measures that automatically checked respondents to help reduce duplicate and fraudulent survey attempts. These prevention measures included checking for duplicate email addresses, cross-checking the reported age and date of birth, and checking attention by asking a single question designed to determine whether the respondent was reading the questions thoroughly. Additionally, once the experiment was completed, a fraud detection analyst reviewed the final data sets, manually checking cases that were flagged as suspicious (eg, multiple respondents with at least an 80% email match and the same IP address). Cases that were deemed to be duplicate or fraudulent responses during this additional review were removed from the data set; these users did not receive an incentive (as indicated in the conditions offering an incentive).

### Measures

The primary outcomes for the study were recruitment time (the length of time [in hours] that Instagram ads ran before reaching 150 total completes), recruitment cost (the amount of money spent on the ad campaign), data collection cost (the amount of money spent on the ad campaign and incentives), response rates (the total number of survey completes and the percentage of eligible respondent completes), and data quality (the percentage of data removed postsurvey after fraud detection measures were taken). In addition to these measures, we analyzed advertising data to determine whether ad delivery across conditions may have impacted the results; these measures included reach (the number of unique users who saw an ad), impressions (the total number of views on an ad, including multiple views from the same user), cost per 1000 impressions (CPM), link clicks (the number of users who clicked on the link attached to the ad), link click-through rate (the number of link clicks divided by the number of impressions), and cost per link click. Lastly, we analyzed participant demographics by condition to help identify any potential sample differences that may have impacted differences in results between conditions, including sex, age, DMA of residence, race/ethnicity, education, and disposable income, as reported in the survey.

### Ethical Considerations

The study protocol was reviewed and approved by the RTI International Institutional Review Board on June 8, 2022 (ID 00022059). All participants gave their informed consent for inclusion before they participated in the online survey. The survey data used for analysis were deidentified. Email addresses were used to distribute incentives but were collected and stored separate from survey responses.

## Results

Table 2 shows the cost to run each condition’s ad campaign, the cost of each data collection (recruitment costs plus incentive costs), amount of time each ad campaign ran, number of screener and survey completes for each condition, and percentage of data removed for fraud for each condition.

Table 3 provides data on the Instagram campaign performance by condition.

Table 4 provides data on the demographics of respondents by condition.

Respondents for all conditions mostly identified as being of White race and female sex. The no-incentive condition had a slightly higher percentage of female and White respondents compared to those in the US $5 and US $15 incentive conditions. The average age of respondents across all three conditions ranged from 19.38 to 20.31 years. Distributions of respondents across DMAs per condition varied, as did educational attainment and disposable income. Approximately one-fifth of respondents in the no-incentive condition reported having more than US $200 to spend freely each week compared to less than one-tenth of respondents in the US $5 and US $15 incentive conditions.

**Table 2:** Main outcomes for each incentive condition.

Outcomes	No incentive	US $5 incentive	US $15 incentive
Recruitment time (hours)	60	39	17
Recruitment ad campaign cost (US $)	1398.23	864.33	338.64
Eligible based on screener, n	55	166	166
Survey completes, n (%)	24 (43.6)	149 (89.8)	155 (93.4)
Surveys deemed fraudulent, n (%)	0 (0)	7 (4.7)	7 (4.5)
Final sample of nonfraudulent survey completes	24	142	148
Cost (US $) per survey complete (ads only)	58.26	5.84	2.38
Cost (US $) per survey complete (including incentives)	58.26	10.84	17.38

**Table 3:** Campaign performance by incentive condition.

Advertising performance measure	No incentive	US $5 incentive	US $15 incentive
Reach, n	44,383	30,799	16,164
Impressions, n	75,299	46,623	19,854
CPM^a^ (US $)	18.57	18.54	17.06
Link clicks/outbound clicks	251	408	303
Link click-through rate, %	0.33	0.88	1.53
CPC^b^ (US $)	5.57	2.12	1.12

^a^CPM: cost per 1000 impressions.

^b^CPC: cost per click.

**Table 4:** Demographics of respondents by incentive condition.

Demographic characteristic			No incentive (n=24)		US $5 incentive (n=142)		US $15 incentive (n=148)
**Sex, n (%)**							
	Male	4 (16.7)		34 (23.9)		35 (23.7)	
	Female	20 (83.3)		108 (76.1)		112 (75.7)	
	Prefer not to answer	0		0		1 (0.01)	
Age (years), mean (SD)			19.38 (2.28)		20.18 (2.11)		20.31 (2.09)
**DMA^a^, n (%)**							
	Atlanta	9 (37.5)		0		0	
	Minneapolis-St. Paul	9 (37.5)		0		0	
	Phoenix (Prescott)	6 (25.0)		0		0	
	Denver	0		22 (15.5)		0	
	Seattle-Tacoma	0		41 (28.9)		0	
	Washington, DC (Hagerstown)	0		79 (55.6)		0	
	Boston	0		0		57 (38.5)	
	Tampa-St. Petersburg	0		0		40 (27.0)	
	San Francisco	0		0		51 (34.5)	
**Race/ethnicity^b^, n (%)**							
	American Indian/Alaska Native	2 (7.7)		3 (1.9)		6 (3.0)	
	Asian or Asian American	4 (15.4)		37 (22.8)		44 (23.5)	
	Black or African American	0		21 (13.0)		14 (8.0)	
	Hispanic/Latino/a/x	2 (7.7)		24 (14.8)		27 (15.5)	
	Pacific Islander/Native Hawaiian	0		2 (1.2)		1 (0.6)	
	White	17 (65.4)		68 (42.0)		77 (44.3)	
	Prefer not to answer	1 (3.8)		6 (3.7)		5 (2.9)	
**Education, n (%)**							
	High school graduate or less	11 (45.8)		51 (35.8)		51 (34.5)	
	Some college, no degree	7 (29.2)		47 (33.1)		52 (35.1)	
	Associate degree	1 (4.2)		7 (4.9)		9 (6.1)	
	Bachelor’s degree	5 (20.8)		33 (23.2)		29 (19.6)	
	Master’s degree	0		3 (2.1)		6 (4.1)	
	Don’t know	0		1 (0.7)		1 (0.7)	
**Income (money available to spend freely each week; US $), n (%)**							
	<25	3 (12.5)		30 (21.1)		25 (16.9)	
	25-50	5 (20.8)		44 (31.0)		35 (23.6)	
	51-100	8 (33.3)		27 (19.0)		37 (25.0)	
	101-150	1 (4.2)		10 (7.0)		14 (9.5)	
	151-200	2 (8.3)		9 (6.3)		8 (5.4)	
	>200	5 (20.8)		11 (7.7)		14 (9.5)	
	Prefer not to answer	0		11 (7.7)		15 (10.1)	

^a^DMA: designated market area.

^b^Question indicated to select all that apply.

## Discussion

### Principal Findings

This study was conducted to analyze the impact of varying incentive amounts on the ability to recruit participants through social media advertisements for an online survey. Ultimately, we observed that a higher incentive amount led to overall quicker data collection with less money spent on ads. Ads in the US $15 incentive condition were able to run for half the amount of time and used less than half the advertising budget needed to generate a similar number of survey completes as the US $5 incentive condition. Further, click-through and completion rates were both slightly higher for the US $15 incentive condition than the US $5 incentive condition (1.53% vs 0.88% and 93.4% vs 89.8%, respectively), which likely contributed to the shorter recruitment period for the US $15 condition. Less than 5% of the data were detected as fraudulent in both the US $5 and US $15 incentive conditions.

It is important to note that although the US $15 incentive condition resulted in the fastest data collection, highest completion rate, and low levels of fraud, the total cost per survey complete (an aggregation of ad spending and cost of the incentive per completion) for this condition was US $17.38 (US $15 for the incentive and US $2.28 for the response), compared to US $10.84 for the US $5 incentive condition (US $5 for the incentive and US $5.84 for the survey response). Although the US $5 incentive condition took longer to achieve complete data collection, the completion rate was still high and fraud detection levels were similarly negligent. Considering this, the US $5 incentive condition should be considered the most cost-effective option. However, other costs associated with longer recruitment campaigns (such as labor for additional monitoring and fraud detection) should be considered based on how the cost of labor is defined within the organization running data collection.

The least successful condition in terms of recruitment cost, time, and the number of total survey completes was the no-incentive condition. Although no fraud was detected (likely due to the lack of monetary motivation), data collection for this condition was extremely costly and slow. Fewer than half the eligible respondents completed the survey and only 0.33% of those who saw the recruitment ad clicked on it, making recruitment a much lengthier and more expensive process, resulting in a cost of US $58.25 per survey complete for only 24 total completes. Due to the extremely low completion rate, lengthy data collection, and expensive cost per complete, recruiting through social media ads for online surveys without offering a monetary incentive is not recommended.

Across the US $5 and US $15 incentive conditions, respondent demographics were relatively consistent. However, of note, those in the no-incentive condition were more likely to be White, female, have a high school education or less, and have more disposable income (defined as more than US $200 to spend freely each week). These findings demonstrate that providing no incentive may result in a less diverse population of respondents, potentially attracting a select group of people willing to take a survey that does not have a monetary reward. Further investigation would be needed to assess whether these socioeconomic status–related variables impact or bias responses.

### Limitations

This study has several limitations. First, we were unable to fully explore the differences in the levels of fraudulent data by condition due to the short timing of each campaign. Fraudulent data typically increase over time as a campaign gets more exposure and ads begin to be shown to the same users multiple times. If the campaigns ran for a longer period of time with a larger allocated budget, we may have been able to detect a greater difference in the level of fraudulent data removed from each sample by condition; future experiments with larger budgets for ad campaigns are suggested to examine this. Second, we were unable to quantify other costs associated with data collection (eg, the labor for monitoring ads and running regular fraud detection) beyond the cost to run the ad campaign on the platform and the total incentive amount. This limitation prevented us from truly understanding the fully comprehensive cost differentials between conditions.

In addition, each campaign was implemented in a different grouping of locations, which could attribute to behavioral or demographic differences between the condition samples. However, we felt it was important to run the campaigns simultaneously and without any audience overlap to help ensure that cost-related factors associated with the auction-based buying of advertisements on Instagram’s platform would not affect our results. For example, ads on Instagram can cost more one day compared to other days simply due to competition for ad space from other advertisers during that time. Additionally, when there is audience overlap between campaigns running at the same time, these campaigns will compete against each other in the auction, driving up the overall costs of campaigns. To note, costs may have been higher in certain DMAs depending on whether other advertisers were trying to target a similar audience at the same time; however, since the CPM was relatively similar across conditions, this instance is unlikely. Lastly, the topic of our survey was generic and included noninvasive questions about general social media use and perceptions of digital ads in an effort to remain neutral for all possible populations responding to the ads. These palatable questions may have resulted in a more enjoyable survey-taking experience—and thus a higher completion rate—compared to surveys that are more focused on public health–related topics or include more difficult or thought-provoking questions. This limitation should be considered when comparing these results to other social media recruitment efforts.

### Conclusions

In conclusion, our results suggest that providing a larger monetary incentive when using social media to recruit participants for an online survey will ultimately result in quicker and cheaper data collection in terms of ad campaign length and spending. Comparatively, providing no incentive at all results in an extremely slow and costly data collection and is therefore not a recommended approach. When considering incentive amounts, a US $5 incentive appears to be more cost-effective than a US $15 incentive; however, this determination does not consider other costs such as labor that might be associated with running a campaign for a longer period of time. Future studies should test conditions with smaller increases in incentives (eg, US $5, $10, $15) to find the ideal amount in terms of overall data collection costs. Future experiments are also suggested to run campaigns for longer periods of time to better understand differences in fraudulent attempts and respondents across conditions.

## Abbreviations

CPM: cost per 1000 impressions
DMA: designated market area

## Data Availability

All data analyzed during this specific study are included in this published article.
